# Supplementary material for: Small extracellular vesicles mediate the antihyperalgesic effect of bone marrow stromal cells: the role of “priming”
Source: bioRxiv. 2026 May 12:2026.05.08.723785. Preprint. [Version 1] doi: 10.64898/2026.05.08.723785 (PMC13192723; doi:10.64898/2026.05.08.723785)

bioRxiv preprint doi: https://doi.org/10.64898/2026.05.08.723785; this version posted May 12, 2026. The copyright holder has placed this preprint (which was not certified by peer review) in the Public Domain. It is no longer restricted by copyright. Anyone can legally share, reuse, remix, or adapt this material for any purpose without crediting the original authors.

Supplementary Fig. 1. Anti-hyperalgesia by serum-derived sEVs/exosomes in mice after tendon injury. **A**. Flowchart of the experiment. BMSCs or culture medium was injected (i.v.) into the TL rats, and exosomes were isolated from the serum of the donor animals. Isolated exosomes were injected into mice at 4 w after TL, and behavioral tests were conducted at 3 h to 7 d after exosome injection. **B**. An IV injection of exosomes (0.02 ml) isolated from BMSC-treated TL animals (BMSC-TL) elevated EF_{50} at the 3 h and 1 d timepoints, indicating attenuation of behavioral hyperalgesia in TL mice as compared to exosomes from culture medium-treated animals (Med-Ex). **C**. BMSC-Ex reduced %Time in the light chamber in CCI mice at 1 d after injection. *, $p<0.05$, **, $p<0.01$, vs. TL-4 w; ###, $p<0.001$, vs. BMSC-TL. N=5/Group. Post-hoc comparisons after one-way or two-way ANOVA with repeated measures.

Supplementary Fig. 2. The effect of BMSC culture-derived sEVs/exosomes on behavioral hyperalgesia in male mice. **A**. Flowchart of the experiment. The BMSC culture was primed with serum (20%, 37°C, 16 hr) from TL or naive rats one week after seeding. Ten milliliters of culture medium were used to isolate exosomes. **B**. An IV injection of exosomes (0.02 ml) isolated from the BMSC culture primed with serum from TL animals (TL-Ex) elevated EF_{50} in CCI mice, compared to exosomes from the BMSC culture treated with naive serum (Naïve-Ex). **C**. TL-Ex reduced % Time in the light chamber in CCI mice at 1d after injection, suggesting a reduced aversion to a noxious stimulus. *, $p<0.05$, **, $p<0.01$, ***, $p<0.001$, vs. CCI-4w; ##, $p<0.01$, ###, $p<0.001$, vs. TL-Ex 1x. N=5/Group. Post-hoc comparisons after one-way or two-way ANOVA with repeated measures.

bioRxiv preprint doi: https://doi.org/10.64898/2026.05.08.723785; this version posted May 12, 2026. The copyright holder has placed this preprint (which was not certified by peer review) in the Public Domain. It is no longer restricted by copyright. Anyone can legally share, reuse, remix, or adapt this material for any purpose without crediting the original authors.

Fig. S1

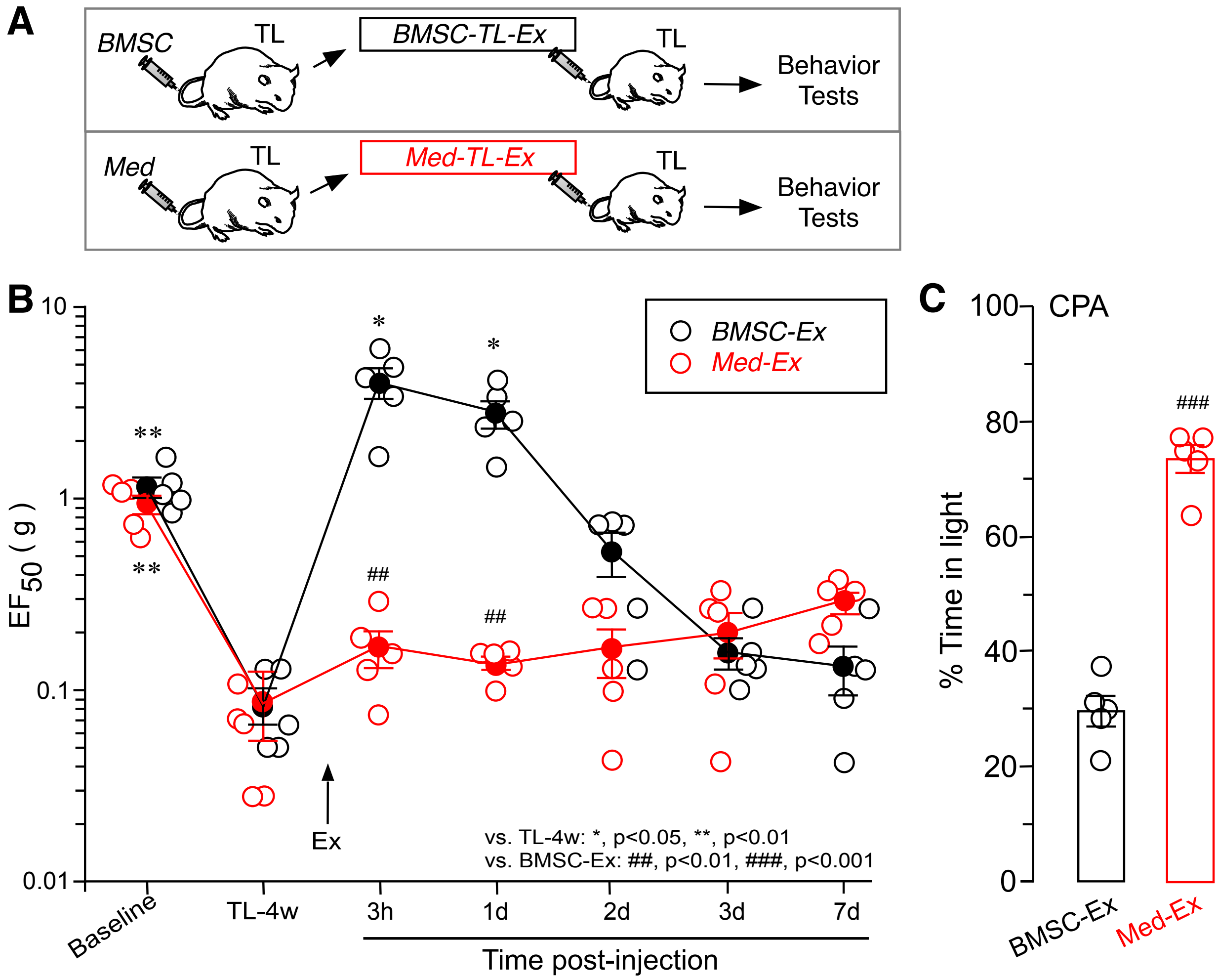

bioRxiv preprint doi: https://doi.org/10.64898/2026.05.08.723785; this version posted May 12, 2026. The copyright holder has placed this preprint (which was not certified by peer review) in the Public Domain. It is no longer restricted by copyright. Anyone can legally share, reuse, remix, or adapt this material for any purpose without crediting the original authors.

Fig. S2

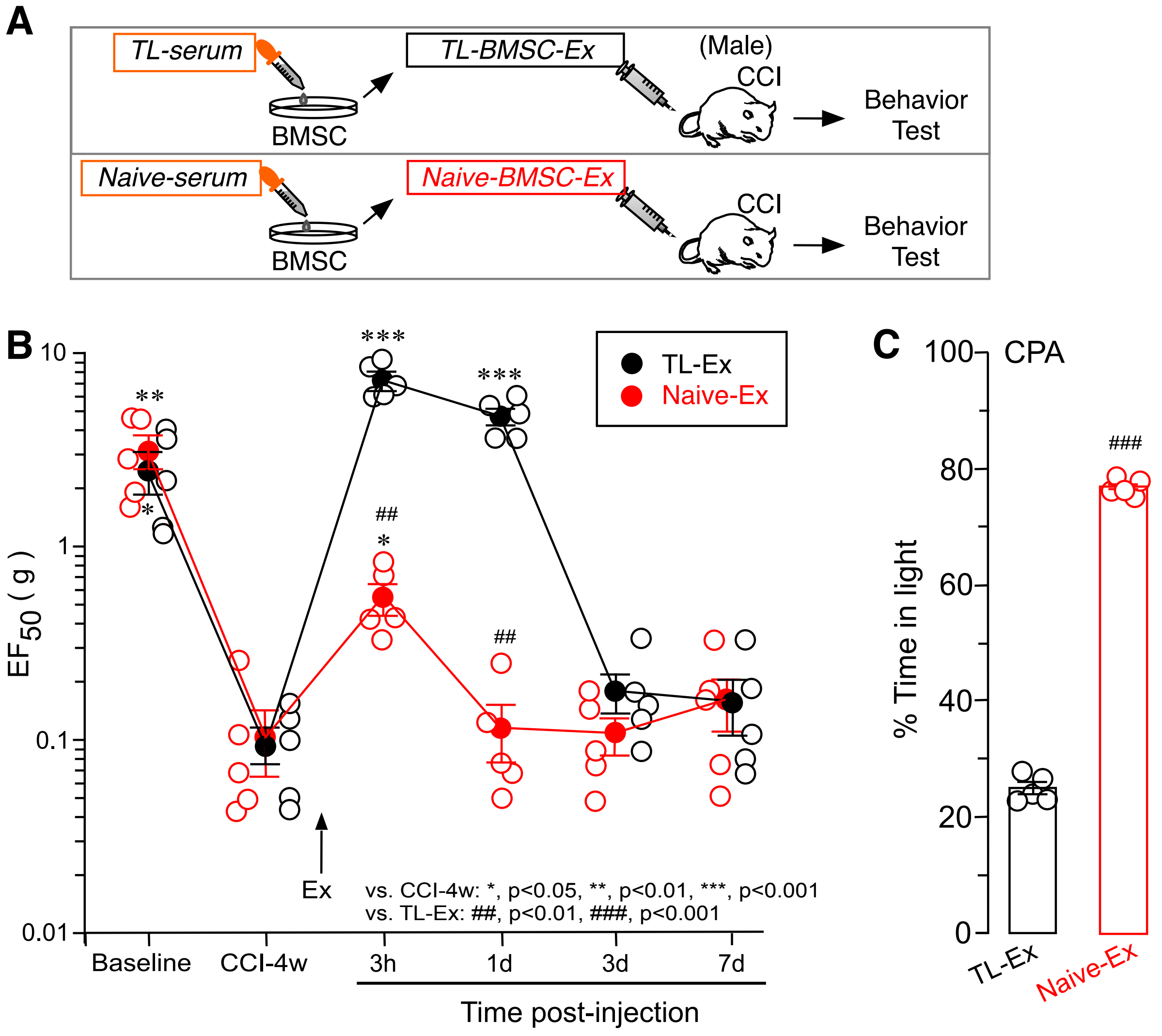

Supplement: 1 [file NIHPP2026.05.08.723785v1-supplement-1.pdf]
